# Exploring the Use of Virtual Funerals during the COVID-19 Pandemic: A Scoping Review

**DOI:** 10.1177/00302228211045288

**Published:** 2021-09-22

**Authors:** Andie MacNeil, Blythe Findlay, Rennie Bimman, Taylor Hocking, Tali Barclay, Jacqueline Ho

**Affiliations:** 1 Factor-Inwentash Faculty of Social Work, University of Toronto, Toronto, Canada; 2 Institute for Life Course and Aging, University of Toronto, Toronto, Canada

**Keywords:** funerals, disenfranchised grief, coping/adaptation, grief, mourning

## Abstract

The COVID-19 pandemic and physical distancing limitations have had a profound impact on funeral practices and associated grieving processes. The purpose of the present scoping review is to summarize the existing literature on the emerging use of virtual funerals. Five medical databases, five social science databases, and five grey literature databases were searched, identifying 1,351 titles and abstracts, of which 62 met inclusion criteria. Four themes, each with various subthemes emerged: (a) Impact of virtual funerals on coping with death; (b) Impact of the COVID-19 pandemic on the funeral industry; (c) Benefits and disadvantages of virtual funerals; and (d) Future implications for health and social work practitioners. Virtual funerals are an evolving resource for individuals, families, and communities to mourn in response to the interruptions to traditional grieving practices due to the COVID-19 pandemic.

The COVID-19 pandemic is a major public health crisis compromising the well-being of individuals, communities, and nations worldwide. COVID-19 is a highly contagious respiratory illness that can be spread through both direct contact (i.e., respiratory droplets, close physical contact) and indirect contact (i.e., contaminated objects, airborne contagion) (Guo et al., 2020; World Health Organization (WHO), 2020a; Zhang et al., 2020). As of August 18, 2021, there have been more than 208 million confirmed cases of COVID-19 and more than 4.3 million COVID-19-related deaths worldwide (WHO, 2021). In the United States alone, there have been over 37 million confirmed COVID-19 cases and approximately 621,000 deaths (Center for Disease Control and Prevention (CDC), 2021).

The COVID-19 pandemic has placed an unprecedented economic strain on global health systems. Given the highly contagious nature of COVID-19, governments are under increasing pressure to reduce the burden of COVID-19 by enacting public health measures to contain the spread of the virus. Governments have imposed physical distancing limitations in an attempt to minimize interactions between people and ultimately reduce disease transmission (Lotfi et al., 2020).

The COVID-19 pandemic has forced many individuals and families to confront the inevitability of death, dying, and loss, while simultaneously causing disruption to the traditions that surround the dying process (Bertuccio & Runion, 2020; Mitima-Verloopa et al., 2021). Traditionally, the experience of losing a loved one is often accompanied by ritualistic and spiritual practices, which serve as a way for people to mourn the loss and begin the grieving process (Mitima-Verloopa et al., 2021; O’Rourke et al., 2011; Wolfelt, 2016). Deaths during the COVID-19 pandemic have been said to exemplify “bad deaths”, which are characterized by physical discomfort, social isolation, and psychological distress, both for the individual and for their family members and friends who cannot be with their loved ones in their final moments (Simpson et al., 2021). The forced separation of patients and their loved ones may also inhibit people from engaging in cultural, religious, or spiritual practices that are part of the dying process, such as prayer in the presence of the dying or deceased and traditional practices of preparing the body. Simultaneously, physical distancing limitations have fundamentally altered traditional social practices and gatherings that would take place after death, such as funerals. Funeral services support the experience of collective grief by offering a platform where expressions of beliefs, thoughts, and feelings are shared (Batesville, 2021; Rumbold et al., 2019; Wolfelt, 2016). Funeral services provide the opportunity for the physical demonstration of support and remembrance of the person who has died, ultimately providing feelings of ongoing support and togetherness (Wolfelt, 2016). Regardless of one’s cultural, religious, spiritual, or personal belief systems, funeral services have been shown to provide a positive space for grief, bereavement, and mourning to take place (Mitima-Verloopa et al., 2021; O’Rourke et al., 2011; Wolfelt, 2021).

The COVID-19 pandemic has fundamentally changed funeral practices on a global scale since its outset (Júnior et al., 2020). The restrictions imposed to curb the spread of the virus have affected people’s ability to host and observe funeral practices as they normally might (Matsuda et al., 2021). In-person funeral gatherings have been linked with increased COVID-19 transmission (Ghinai et al., 2020), and many regions have enforced size limits on gatherings to minimize the spread of COVID-19. Additionally, the CDC recommends against many practices that would traditionally take place at funerals, such as sharing objects, including significant religious books, and hugging and kissing, due to the need to maintain a distance of six feet from others (CDC, 2020).

Physical distancing limitations have led grieving families and funeral providers to face a limited set of perhaps unfavorable options, including hosting funerals online, restricting funerals to very small in-person and/or outdoor gatherings, or deciding to postpone funerals until after restrictions are eventually lifted (CDC, 2020). Funeral directors have been overwhelmed in trying to meet new restrictions and facilitate appropriate memorials, while also struggling to meet the demands of the unprecedented waves of deceased bodies to care for resulting from the high death toll enacted by COVID-19 (Lehmann, 2020).

Evidence already exists of the pandemic’s adverse impact on mental health and emotional well-being (Júnior et al., 2020). The consequences that these altered, limited funeral practices will have on mental health, well-being, and bereavement outcomes, including reactions such as disenfranchised grief (Doka, 1989) and complicated grief (Shear, 2015), are not yet fully understood. In light of the known importance of grieving and funeral rituals, their ongoing absence prevents people from having a “chance to say a final goodbye” (Júnior et al., 2020), and subsequently may have serious impacts on the mental health of those experiencing loss (Canadian Press, 2020; Júnior et al., 2020).

Given the limited knowledge on the use of virtual funerals during the COVID-19 pandemic, the purpose of this scoping review is:

1. To summarize the available literature on the impact of virtual funerals on the grieving process during the COVID-19 pandemic.
2. To categorize the available literature on the impact of the COVID-19 pandemic on the funeral industry.
3. To describe the various benefits and disadvantages of virtual funerals.
4. To examine the implications of virtual funeral practices for healthcare practitioners in supporting their clients during the grieving process.

## Methods

The present scoping review utilized the method outlined by Arksey and O’Malley (2005) to synthesize and analyze a wide range of relevant literature on the use of virtual funerals during the COVID-19 pandemic and to identify gaps in existing literature on this topic.

A computerized search of the literature was conducted in January and February 2021. A total of 15 databases, including 5 medical databases, 5 social science databases, and 5 grey literature databases were searched: Medline (2019—), Embase (2019—), CINAHL (2019—), World Health Organization COVID-19: Global Literature on Coronavirus Database (2019—), Cochrane COVID-19 Study Register (2019—), Social Work Abstracts (2019—), Sociological Abstracts (2019—), International Bibliography of Social Sciences (2019—), AgeLine (2019—), PsycINFO (2019—), Google Scholar (2019—), New York Academy of Medicine’s Grey Literature Database (2019—), OpenGrey Literature Database (2019—), US Newsstream (2019—), and Canada Newsstream (2019—). A manual search through reference lists in the reviewed articles was also conducted.

### Search Strategies

A comprehensive list of search terms was generated related to the following three concepts: funerals, online, COVID-19. The terms for COVID-19 were drawn from a recent Cochrane review (Krishnaratne et al., 2020). Please see Online Appendix A for all keywords and subject headings used in these searches.

### Inclusion and Exclusion Criteria

Peer-reviewed and grey literature was considered appropriate for inclusion in this study if significant sections of the article focused on the use of virtual funerals during the COVID-19 pandemic. This includes studies that have gathered original data (quantitative or qualitative), as well as commentaries, perspectives, opinion pieces, and news articles. Articles published anywhere in the world were eligible for inclusion, however, articles that were not available in English were excluded. Data parameters were set from December 2019 to February 2021 based on when the first research on the COVID-19 pandemic was published.

### Data Extraction

**Figure 1.**
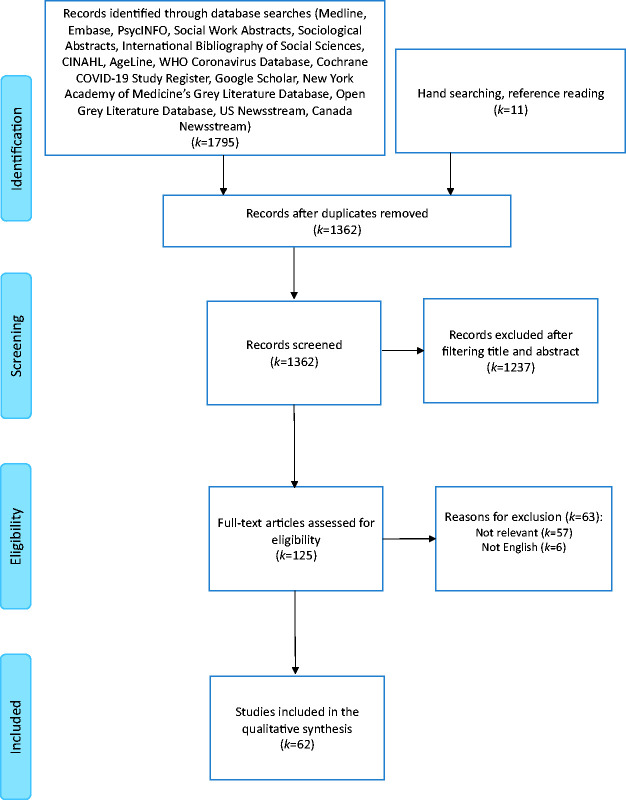
Flow diagram for the identification of articles, inclusion, and exclusion assessment.

As presented in Figure 1, the database searches resulted in 1,351 unique titles and abstracts. 11 additional sources were identified through hand searching and reference reading, resulting in a total of 1,362 unique titles and abstracts. Two evaluators then screened each title and abstract independently. In a case of discrepancy between the two evaluators during the title and abstract screening, the article was included in the full-text review to determine eligibility. This initial title and abstract screening identified 125 articles that were appropriate for full-text review. There were 1,237 sources that were excluded during the screening process because their title and abstract indicated they were not relevant to the current scoping review, as their subject matter was not focused on the use of virtual funerals during the COVID-19 pandemic. After reviewing the full text of the 125 articles, a total of 62 articles met the inclusion criteria and were selected for data extraction. There were 63 article that were excluded after the full-text review because their content was not relevant to the current scoping review (k = 57) and because they were not published in English (k = 6).

## Results

A total of 62 academic and grey literature articles were identified for review (See Table 1). Four main themes emerged from the literature:

**Table 1.** Data From Articles Included in the Review (N = 62).

Author(s)	Type of reference	Themes
Adelman (2020)	Journal Article – Commentary	B1, B4, C2, C3
Alaimo (2020)	News Article	B1, B3, C4, C6
Aleccia (2020)	News Article	B1, B2, C1, C2, C6
Arévalo Aldea del Rey (2020)	Journal Article – Commentary	B1, B2, B3, C1, C4
Bitusikova (2020)	Journal Article – Commentary	A1, A2, B1, B4, C2, C4, C5
Booth (2020)	News Article	A1, C1, C4, C6
Boyer-Dry (2020)	News Article	A1, A2, C2, C3, C4, C5
Brown (2020)	News Article	B1, B2, B4, C2, C4, C6
Burger (2020)	News Article	A1, A2, B1, C1, C2, C3, C4, C5, C6
Burrell & Selman (2020)	Journal Article – Mixed Methods Review (N = 17)	A1, A2, B1, B2, B4, C1, C3, C4, C5
Cann & Hoy (2020)	Commentary	A1, A2, C4
Cann et al. (2020)	Practitioner Resource	A1, A2, B1, C1, C2, C3, D
Carr et al. (2020)	Journal Article – Commentary	A1, B1, C1, C3, D
Centers for Disease Control and Prevention (2020)	Government Guidelines	A1, A2, C1, C3
Christman (2020)	News Article	A1, A2, B1, B2, B3, B4, C1, C3, C4, C6
Corpuz (2020)	Journal Article – Commentary	A1, B1, C1, C5
Corpuz (2021)	Journal Article – Commentary	A1, A2, B1, B3, B4, C5
Egan-Elliott (2020)	News Article	B1, B3, C1, C4, C6
Evans et al. (2020)	Report	A1, A2, D
Felter et al. (2020)	News Article	A1, A2, B1, C1, C2
Garcia (2020)	News Article	C1, C4, C6
Goldsmith (2020)	News Article	C2, C4, C5
Goveas & Shear (2020)	Journal Article – Commentary	A1, B1, D
Greek (2020)	Journal Article – Letter to the Editor	C2, C4
Johns et al. (2020)	Journal Article – Brief Note	A1, B2, B4, C1, C4, D
Kantor (2020)	News Article	A1, A2, B1, B2 C1, C2, C4, C5, C6
Kelley (2020)	News Article	C1, C2, C4, C6
Kelly (2020)	News Article	A1, B1, B4, C1, C2, C3
Kernan (2020)	News Article	C1, C3, C6
Ketcham (2020)	News Article	B2, C1, D
Kuyenhoven (2021)	Blog Post	A1, A2, C1, C2, C4, C5, C6
Langlois (2020)	News Article	A1, B1, B4, C1, C2, C4, C5, C6
Li (2020)	Magazine Article	A1, A2, B1, C5
Lockwood (2020)	News Article	B2, C1, C6
Lowe et al. (2020)	Journal Article – Rapid Perspective Review	A1, A2, B1, C1, C2, C6, D
Mackenzie (2020)	News Article	A1, A2, C1, C2, C3, C4, C5
Maitra (2020)	Journal Article – Commentary	A1, A2, B1, C2, C4
Miller (2020)	News Article	A2, C2, C4
Murphy (2020)	News Article	B1, B4, C1, C2, C3
Muturi et al. (2020)	Journal Article – Commentary	A2, B1, C1, C2, C3, C4, C5
Parsons (2020)	News Article	A2, C1, C6
Patterson (2020)	News Article	A1, C1, C5
Pauly (2020)	News Article	A1, A2, B2, C1, C2
Petry et al. (2020)	Journal Article – Commentary	A1, A2, B1, C1, C4, D
Prior (2020)	News Article	A1, A2, C1, C2, C3
Rahman (2020)	News Article	C1
Rentsch (2020)	News Article	C1, C2, C3, C4, C5, C6
Schwartz (2020)	News Article	A1, A2, B1, C2, C3, C4, D
Schuck et al. (2020)	News Article	A1, A2, B1, B4, C1, C2, C4
Selman et al. (2020)	Journal Article – Mixed Methods Review	A1, B1, D
Serbin (2020)	News Article	C2, C4, C6
Shoji & Matsue (2020)	Working Paper	A1, B1, B4, C1
Shtotland (2020)	News Article	A1, A2, B1, C1, C2, C3, C4
Sims (2020)	News Article	A2, B2, C1, C2, C5, C6
Sivell (2020)	News Article	A1, B4, C2, C4
Tsai (2020)	News Article	A1, B1, C1, C3, C4, C5, C6
Wallace et al. (2020)	Journal Article – Commentary	A1, A2, B1, C1, C3, D
Walsh (2020)	News Article	A2, B2, B3, B4, C1, C2, C4
Waters (2020)	News Article	A1, A2, C1, C2, C3, C4
Wilensky (2020)	News Article	A2, B1, B4, C1, C2, C3
Wood (2020)	Magazine Article	B1, B4, C1, C2, C3, C4
World Health Organization (2020b)	Guidelines	B1, B4, C1, C3

1. Impact of virtual funerals on coping with death
2. Impact of COVID-19 pandemic on the funeral industry
3. Benefits and disadvantages of virtual funerals.
4. Future considerations for health and social work practitioners

Included articles are mapped to main themes and subthemes in Table 1. The coding legend is provided in Table 2.

**Table 2.** Coding Legend of Main Themes and Subthemes Identified in the Review.

Impact of virtual funerals on coping with death	
Grief responses	A1
Meaning-making	A2
Impact of the COVID-19 pandemic on the funeral industry	
Required modification of rituals	B1
Increased demands on the funeral industry	B2
Mental health of workers	B3
Inaccess to sacred spaces	B4
Considerations for the use of virtual funerals	
Restrictions on size of gatherings and physical distancing rules	C1
Preferred platforms and impact on the funeral industry	C2
Tech accessibility	C3
Increased reach	C4
Provides opportunity to grieve	C5
Choosing to delay the funeral indefinitely	C6
Future considerations for health and social work practitioners	D

### Impact of Virtual Funerals on Coping with Death

The impact of virtual funerals on how bereaved individuals cope with death was a prevalent theme throughout the literature and was identified in 41 of the 62 articles. This theme was categorized into two subthemes: grief responses and meaning making.

### Grief Responses

The literature frequently reported that the use of virtual funeral practices during the COVID-19 pandemic has fundamentally changed how people grieve (Burrell & Sellman, 2020; Carr et al., 2020; Corpuz, 2020, 2021; Goveas & Shear, 2020; Johns et al., 2020; Maitra, 2020). The inability to gather in-person and collectively mourn creates challenges for people to process their grief in traditional ways (Carr et al., 2020; Johns et al., 2020; Maitra, 2020). The absence of familiar rituals may lead to complicated grief responses, such as prolonged grief (Goveas & Shear, 2020; Johns et al., 2020; Wallace et al., 2020) and disenfranchised grief (Cann et al., 2020; Petry et al., 2020; Wallace et al., 2020). Furthermore, because COVID-19 fatalities often exemplify characteristics that are indicative of a “bad death,” such as physical discomfort and social isolation, bereaved loved ones may experience heightened feelings of guilt and anger throughout the grieving process (Carr et al., 2020).

In addition to complicated grieving experiences, bereaved families have also been deprived of the simple practices that would traditionally bring comfort during a time of mourning, such as physical touch with loved ones (Goveas & Shear, 2020; Petry et al., 2020; Shoji & Matsue, 2020). These challenges are compounded by the broader social isolation and psychological distress that characterize the COVID-19 pandemic (Evans et al., 2020; Goveas & Shear, 2020). For many bereaved individuals, the grieving process has been disrupted by external stressors related to COVID-19, such as financial stress, precarious employment, and concerns over their health and the health of their loved ones (Carr et al., 2020; Wallace et al., 2020).

### Meaning Making

The literature described how people have found ways to make virtual funeral practices a meaningful experience, even in the absence of physical closeness (Bitusikova, 2020; Burrell & Selman, 2020; Cann & Hoy, 2020; Corpuz, 2021; Evans et al., 2020; Maitra, 2020; Muturi et al., 2020; Petry et al., 2020; Wallace et al., 2020). Physical distancing limitations have forced bereaved families to adopt innovative online rituals to support the grieving process (Corpuz, 2021; Lowe et al., 2020). The literature highlights that what constitutes a meaningful funeral is subjective, and it is essential that people find meaning in a way that feels appropriate to them (Burrell & Selman, 2020). Many bereaved individuals are modifying traditional funeral practices through virtual means, highlighting the numerous ways to make a meaningful experience during the grieving process. For example, one individual described how a virtual shiva became a meaningful way to commemorate their loved ones because it reached a wider audience of extended friends and family than would be possible for an in-person service (Bitusikova, 2020). Another family found meaning in the process of planning their online event, enjoying the experience of scanning photos to be shared during a virtual slideshow (Mackenzie, 2020). Although virtual funerals may not replace certain elements of in-person funerals, such as physical touch, they can still be a meaningful experience that provides a sense of social connectedness for those who are grieving (Bitusikova, 2020; Mackenzie, 2020; Maitra, 2020; Selman et al., 2020).

#### Impact of the COVID-19 Pandemic on the Funeral Industry

The impact of the pandemic on the funeral industry and staff was the second major theme identified in the literature and was recorded in 42 out of the 62 articles. This theme was further categorized into four subthemes: required modification of rituals, increased demands on the funeral industry, mental health of service professionals, and inaccess to sacred spaces.

### Required Modification of Rituals

The impact of COVID-19 on rituals associated with the funeral industry was frequently cited within the literature (Aleccia, 2020; Egan-Elliott, 2020; Ketcham, 2020; Walsh, 2020). Funeral rituals are symbolic practices that helps individuals and families process their emotional, physical, and spiritual responses to loss (Carr et al., 2020; Schwartz, 2020). Public health restrictions (i.e., physical distancing) have forced families to forgo ideals of traditional death rituals and move toward virtual funerals (Christman, 2020; Elliot, 2020; Lockwood, 2020; Walsh, 2020). This has led to a profound impact on the funeral service industry and has changed the landscape of funeral rituals (Alaimo, 2020; Egan-Elliott, 2020; Schwartz, 2020). For example, one individual described their experience attending a virtual shiva and its contrast with a traditional in-person shiva (Bitusikova, 2020). Traditional shiva customs, such as washing hands, lighting candles, and covering mirrors, had to be done by each attendee individually in their own home, rather than in a shared space, however the virtual shiva still provided a sense of closure and connectedness (Bitusikova, 2020). Similarly, a Rabbi described live-streaming a burial because the children of the deceased lived in another country and would not be able to attend an in-person funeral due to travel restrictions (Schwartz, 2020). The examples highlight some of the many ways that funeral practices are being virtually modified due to COVID-19.

### Increased Demands on the Funeral Industry

The number of deaths resulting from the pandemic has led to an increase in the demand for funeral service professionals (Arévalo Aldea del Rey, 2020; Brown, 2020). The increased service demands have resulted in the funeral industry encouraging the use of virtual services as a preventive measure to avoid further burdening an already overwhelmed system (Arévalo Aldea del Rey, 2020; Brown, 2020; Egan-Elliott, 2020). In addition, funeral service professionals are also under increasing pressure to reduce the amount of in-person funerals in an effort to avoid unnecessary human contact and potential virus spread (Brown, 2020; Egan-Elliott, 2020). Due to protective measures imposed by public health and the increase of volume and service use, funeral professionals have been forced to be creative in the ways they provide comfort and support to grieving families (Brown, 2020).

### Mental Health of Funeral Service Professionals

The mental health and well-being of funeral directors and other funeral service professionals was frequently noted in the literature reviewed (Alaimo, 2020; Christman, 2020; Walsh; 2020). The mental health of funeral service workers has been impacted by their concerns over potential exposure when preparing the bodies of those who died of COVID-19, as well as the tremendous workload resulting from the increased death rate (Arévalo Aldea del Rey, 2020). This has forced funeral professionals to modify the traditional ways in which they offer support and express compassion to grieving families (Aleccia, 2020; Christman, 2020; Walsh, 2020). Funeral service professionals have born significant responsibility in supporting families through the ongoing social gathering limitations (Alaimo, 2020). With the push towards virtual services, many funeral service professionals have described how distressing it can be to inform bereaved families that they have to make an often undesirable shift to online formats (Alaimo, 2020; Christman, 2020; Walsh, 2020).

### Inaccess to Sacred Spaces

Many articles commented on how the COVID-19 pandemic has removed access to sacred spaces due to the implementation of public health restrictions (Brown, 2020; Christman, 2020; Corpuz, 2021; Sivell, 2020; Walsh, 2020). The inaccess to traditional sacred spaces has greatly impacted how individuals process the loss of a loved one and experience the funeral service itself (Goveas & Shear, 2020). Many individuals have expressed a deepened level of sadness resulting from the inability to grieve in spaces like churches, synagogues, mosques, and temples (Corpuz, 2021; Goveas & Shear, 2020; Wilensky, 2020).

#### Benefits and Disadvantages of Virtual Funerals

The third theme identified in the literature was benefits and disadvantages of virtual funerals. This theme was found in 59 out of the 62 articles reviewed, and included six subthemes: restrictions on size of gatherings and physical distancing rules, preferred platforms and impact on the funeral industry, tech accessibility, increased reach, provides opportunity to grieve, and choosing to delay indefinitely.

### Restrictions on Size of Gatherings and Physical Distancing Rules

The reviewed literature often described how virtual funerals have emerged as a necessary consequence of gathering restrictions and physical distancing limitations (Aleccia, 2020; Booth, 2020; Burrell & Selman, 2020; Johns et al., 2020; Lowe et al., 2020; Shoji & Matsue, 2020). Guidelines from the World Health Organization (WHO) (2020b) and the Centers for Disease Control and Prevention (CDC) (2020) emphasized the need for restrictions on the size of all gatherings, including funerals, to minimize the spread of COVID-19 (WHO, 2020b; CDC, 2021). Many countries around the world, including Canada, Australia, United Kingdom, United States and Brazil, have adopted a 10-person gathering limit for at least part of the pandemic period (Lowe et al., 2020). Those who are able to attend in-person must comply with physical distancing and screening measures for COVID-19 symptoms or travel history prior to entry (Muturi et al., 2020). In the event of unavoidable physical contact, proper infection control, sanitization and personal protective equipment (PPE) must be maintained (Muturi et al., 2020). These restrictions have led some families to choose virtual funerals while others have chosen to delay the formal funeral indefinitely.

### Preferred Platforms and Impact on the Funeral Industry

The literature in this scoping review highlighted some common platforms that people turned to for hosting online funerals. Zoom (Adelman, 2020; Boyer-Dry, 2020; Felter et al., 2020; Goldsmith, 2020; Kantor, 2020; Mackenzie, 2020; Schwartz, 2020; Wilensky, 2020; Wood, 2020), Facebook Live (Brown, 2020; Kelley, 2020; Miller, 2020; Rentsch, 2020; Sims, 2020; Sivell, 2020), and YouTube Live (Kelly, 2020; Langlois, 2020; Muturi et al., 2020; Waters, 2020) were the most common streaming platforms used for online funeral services. Zoom, YouTube Live, and Facebook Live were described as easy to use and access, with the added benefit of having zero cost. Alternatively, some people have turned to pre-existing services provided by websites such as eCondolence.com (Aleccia, 2020), Shiva.com (Aleccia, 2020), GatheringUs.com (Boyer-Dry, 2020; Cann et al., 2020; Muturi et al., 2020), OneRoom (Muturi et al., 2020; Waters, 2020), and TribuCast (Muturi et al., 2020; Waters, 2020), all of which involve various pricing plans. Virtual funerals will undoubtedly continue even when in-person services resume, as they have had unexpected benefits, such as reaching more people and reducing anxiety around close physical contact with others (Lowe et al., 2020). This shift will have long-lasting implications for the funeral industry, as some service users may only hire funeral homes that can offer live streaming to ensure a wider audience can be reached, even after physical distancing limitations are lifted (Aleccia, 2020).

### Tech Accessibility

Opinions vary regarding the effectiveness of online funerals for supporting the grieving process. Proponents of the practice trumpet the short time needed to plan and execute an event, and the ease of attending a funeral from the comfort of one’s own home, without needing to travel (Boyer-Dry, 2020; Muturi et al., 2020; Rentsch, 2020; Waters, 2020). Others posit that services do not feel the same, or enable as genuine a supportive emotional connection, as being physically present (Burrell & Selman, 2020; Cann & Hoy, 2020; Carr, 2020; Kelly, 2020; Tsai, 2020). Resources and suggestions, like funeral facilitators acknowledging and thanking virtual attendees for their online presence during the service, have emerged to help this practice succeed (Burrell & Selman, 2020). Some note that the effectiveness of virtual funerals fluctuates, often depending on the needs and specific family dynamics of mourners (Burger, 2020). Some may choose not to have virtual funerals because they do not fully understand the concept (Christman, 2020).

However, the literature predicts that as awareness of virtual funerals increases, there will be a continued rise in the use of virtual funerals, whether implemented by funeral home staff or families themselves (Cann & Hoy, 2020; Muturi et al., 2020; Schwartz, 2020). Some posit that the accessibility of virtual funerals makes some traditional religious and cultural rituals easier to carry out, including the Muslim tradition of burial within 24–48 hours (Wood, 2020). However, others question the authenticity and spiritual validity of rituals carried out online, such as the Jewish convention of a quorum of 10 to say the traditional mourner’s prayer (Carr, 2020; Wood, 2020). Accessing virtual funerals can be challenging for people who are not savvy with technology, which is often the case for older adults (Carr, 2020; Kelly, 2020; Shtotland, 2020). However, formal and informal caregivers, such as healthcare staff in care facilities and/or younger family members, may be able to support these individuals in connecting (Carr, 2020). Glitches with technology can also pose challenges, although platforms that cater specifically to virtual funerals can prevent and remedy these problems (Muturi et al., 2020; Shtotland, 2020; Tsai, 2020). Virtual funerals have also been praised for their cost-effectiveness and practicality, particularly given the widespread availability of Internet services (Muturi et al., 2020). Virtual funerals are a low-risk option that can prevent the further spread of COVID-19, and offer a means for the bereaved to share a person’s story despite pandemic restrictions (Centers for Disease Control and Prevention, 2021; Rentsch, 2020). Additionally, some feel that virtual funerals have enabled people to be more freely emotional than during in-person services, due to the opportunity to turn off video and audio input, and allow privacy (Waters, 2020; Wood, 2020). Unfortunately, not all funeral homes have adequate technology in place to conduct a virtual funeral, posing a major challenge (Cann & Hoy, 2020).

### Increased Reach

In many circumstances, virtual funerals can reach more people than in-person funeral services, even in pre-pandemic times, were able to. Large numbers of guests can be accommodated (Boyer-Dry, 2020; Kantor, 2020; Shtotland, 2020; Tsai, 2020), particularly as virtual platforms often have no limits on numbers, unlike the constraints of a physical space (Alaimo, 2020; Bitusikova, 2020; Muturi et al., 2020; Wood, 2020). Virtual funerals have offered an alternative for attendees who would otherwise have been unable to attend due to distance, travel costs, illness, self-isolation, immunocompromisation, finances, work obligations, or other challenges (Bitusikova, 2020; Kantor, 2020; Muturi et al., 2020; Schwartz, 2020; Waters, 2020; Wood, 2020). Some report that virtual funerals still make guests feel as though they are actually present in person (Boyer-Dry, 2020; Burrell & Selman, 2020; Egan-Elliott, 2020). Virtual funerals have also provided opportunity for Muslim women, and women from other cultures, who may not traditionally attend some funeral services, to be present (Burrell & Selman, 2020; Wood, 2020). Virtual funerals can be a supportive solution enabling the bereaved to still gather, from all over the world, despite pandemic restrictions like physical distancing and travel bans (Arévalo Aldea del Rey, 2020; Christman, 2020; Garcia, 2020; Johns et al., 2020; Rentsch, 2020; Waters, 2020). The literature notes that the concept of virtual funerals is not new, but has increased since the pandemic, and the increased reach it offers in light of isolating COVID-19 restrictions is a reason for its spike in popularity (Burger, 2020; Egan-Elliott, 2020; Kelley, 2020; Miller, 2020; Rentsch, 2020; Serbin, 2020).

### Provides Opportunity to Grieve

In the midst of COVID-19 restrictions prohibiting the gathering of families and friends, many are turning to virtual platforms as an alternative space to grieve in place of traditional in-person funerals (Lowe et al., 2020). Expressions of grief and support from friends and family, although now through a virtual space, provide closure and the human connection and interaction necessary to counter possibilities of prolonged or complicated grief (Burger, 2020; Corpuz, 2020; Lowe et al., 2020). Rather than postponing a funeral, mourners may benefit from marking the loss of their loved ones and sharing their grief sooner, rather than later (Burger, 2020). As physical distancing restrictions continue, people are inventing new ways to say goodbye, many of whom are focusing less on the physical bodies of the deceased and more on the memorialization of their loved ones (Kantor, 2020).

### Choosing to Delay the Funeral Indefinitely

As a result of the restrictions on the size of gathering, many families are choosing to limit small burial or cremation viewings to close family, in keeping with the pandemic-related regulations on small gatherings. They then choose to postpone public memorial services or ceremonies to a later date when the pandemic is under control (Booth, 2020; Egan-Elliott, 2020; Kelley, 2020; Kernan, 2020; Langlois, 2020; Lockwood, 2020) in hopes of providing a ‘proper’ goodbye that can include larger number of grievers in-person.

#### Future Considerations for Health and Social Work Practitioners

The final theme prevalent in the literature highlighted future considerations for health and social work practitioners. This theme was identified in 11 of the 62 reviewed articles.

The impact of the pandemic on bereaved people’s mental health will have long-lasting implications for relevant client-facing sectors including health care providers and other allied health professionals. Prolonged Grief Disorder (PGD), as well as other forms of grief such as ambiguous loss, complicated, disenfranchised, and collective grief, add nuances to the bereavement process, and ultimately, to care provision. Health and mental healthcare providers, including doctors, palliative care specialists, social workers, and psychotherapists will need to be aware of, and sensitive to, the associated suffering that has accompanied the new type of grief that has spiked during the pandemic. Such examples of suffering include impaired physical health and reduced quality of life, increased suicidality, substance use issues, relationship challenges, and mental health issues including heightened depression and anxiety (Goveas & Shear, 2020; Wallace et al., 2020). These considerations require policy changes, including improved access to, and funding for, psychotherapy services and bereavement counselling (Goveas & Shear, 2020; Petry et al., 2020). Healthcare providers will need to continue supporting patients and their families, helping navigate visitation restrictions and connecting them virtually in order to provide holistic and communicative care (Selman et al., 2020). Similarly to funeral services, healthcare will also continue to be provided through web-based telemedicine, a shifting landscape that must be incorporated into the “new normal” of caring for those who are grieving as a result of the pandemic (Carr et al., 2020).

## Limitations

The present scoping review gathered a substantial amount of relevant works on the topic of virtual funerals, however, these findings should be interpreted in light of some limitations. Due to the fast-evolving nature of the COVID-19 pandemic, new literature is constantly being published, and it will be important to periodically re-evaluate the effects of this phenomenon. It will also be valuable for further research to examine how the use of virtual funerals during the COVID-19 pandemic, as well as its impact on the grieving process and the funeral industry, may vary across geographical, cultural, and religious boundaries. Additionally, although this review has highlighted some immediate impacts of virtual funerals on grieving and bereavement, it is too early to know the lasting implications of virtual funerals on bereavement outcomes. It is important for research to continuously evaluate the effects of COVID-19 on the grieving process and the mental health of those who have lost a loved one during the pandemic. Finally, the present scoping review only included works published in English, which raises concerns over potential bias.

## Conclusion

The COVID-19 pandemic has had a significant impact on the use of virtual funerals and associated grief experiences. Several themes emerged that highlighted some common and shared experiences among those involved in funerals, whether attending or facilitating, throughout this unprecedented time.

The first theme describes how virtual funerals have fundamentally changed the ways in which people cope with death. In the absence of traditional funeral practices, many bereaved individuals and families have been forced to reconcile uncomfortable and unfamiliar changes in grief responses. However, the adoption of virtual funeral practices has also highlighted the resilience of families and communities during the grieving process, and the ability to find new avenues of meaning despite the inability to gather in-person.

As evident in the second theme, the increased dependence on the use of virtual funerals have posed greater challenges on the funeral industry. Increased mortality rates and public health restrictions have pressured funeral service professionals to be adaptable in their approaches to offering the traditional supportive services.

The third theme explored the various benefits and disadvantages to consider when implementing virtual funerals. Opinions vary regarding the effectiveness of using technology to host funerals, ranging from praise about its accessibility and low price, to concerns regarding its strain on emotional connection. Proponents of virtual funerals laud their ability to reach large, global audiences who could not otherwise attend. The literature revealed that the most commonly used platforms for hosting virtual funerals were Zoom, Facebook Live, and YouTube Live, all of which were described as easy to use. In light of the changing reality of funerals during the COVID-19 pandemic, many are navigating newfound ways to seek closure and memorialize their loved ones, whether that be choosing to delay the funeral until COVID-19 is no longer a threat, or to move to a virtual platform due to restrictions on size of gatherings and physical distancing rules. As virtual funerals continue to grow in popularity, many appreciate the comfort of human connection from friends and families that, despite physical distancing measures, has transcended the virtual space. It is projected that for the foreseeable future, the popularity of this practice will only continue to grow.

The final theme that emerged was future considerations and recommendations for relevant practitioners serving impacted populations. The changes to the grieving process as a result of the COVID-19 pandemic, particularly grief experienced as prolonged, ambiguous, complicated, or disenfranchised, require a unique response from care and service providers. Moving forward, professionals in the health and mental health fields, such as doctors, nurses, palliative care specialists, social workers, and psychotherapists, must be prepared to support the unique trauma and grief responses that have resulted from this global public health crisis. Although life will undoubtedly return to a more “normal” state through vaccine programs and herd immunity, the mental health impacts as a result of the pandemic will have long-lasting and enduring effects that require attention.

## Supplemental Material

sj-pdf-1-ome-10.1177_00302228211045288 - Supplemental material for Exploring the Use of Virtual Funerals during the COVID-19 Pandemic: A Scoping Review Click here for additional data file. Supplemental material, sj-pdf-1-ome-10.1177_00302228211045288 for Exploring the Use of Virtual Funerals during the COVID-19 Pandemic: A Scoping Review by Andie MacNeil, Blythe Findlay, Rennie Bimman, Taylor Hocking, Tali Barclay and Jacqueline Ho in OMEGA—Journal of Death and Dying
