# Supplementary material for: Exploring the Use of Virtual Funerals during the COVID-19 Pandemic: A Scoping Review
Source: Omega (Westport). 2021 Sep 22;88(2):425–48. doi: 10.1177/00302228211045288 (PMC10647914; doi:10.1177/00302228211045288)
Supplement: sj-pdf-1-ome-10.1177_00302228211045288 - Supplemental material for Exploring the Use of Virtual Funerals during the COVID-19 Pandemic: A Scoping Review [file sj-pdf-1-ome-10.1177_00302228211045288.pdf]

Appendix A: Search Strategy

<table>
<tr><td>Text Words (used in all databases)</td><td colspan="3">funeral* or memorial* or celebration of life or wake or vigil or burial* or cremat* or shiva or service or antyesti or janazah

online or internet or social network or webcast or video conferenc* or WeChat or social media or Facebook Live or digital* or livestream* or Zoom or web or stream* or virtual* or cyber* or skype or Instagram live or Instagram* or facetime or www

coronavirus or coronavirus infections or COVID-19 or severe acute respiratory syndrome coronavirus or 2019 nCoV or 2019nCoV or 2019-novel or corona vir* or coronavir* or neocorona vir* or neocoronavir or COVID or COVID19 or nCoV 2019 or nCoV 19 or *SARS-CoV-2* or SARS-like coronavirus severe acute respiratory syndrome coronavirus-2</td></tr>
<tr><td>Time and Date of Search</td><td>Database</td><td>Subject Headings Specific to Database</td><td>Results</td></tr>
<tr><td>Jan 31, 2021 8:00pm</td><td>Medline (Ovid)</td><td>Funeral rites
Burial
Cremation
Death rites
Online social networking
Internet
Social media
SARS-CoV-2
COVID-19</td><td>105</td></tr>
<tr><td>Jan 31, 2021 8:00pm</td><td>PsychINFO (Ovid)</td><td>Funeral rites
Burial
Cremation
Death rites
Online social networking
Internet
Social media
SARS-CoV-2
COVID-19</td><td>13</td></tr>
<tr><td>Jan 31, 2021 8:00pm</td><td>Embase (Ovid)</td><td>Funeral rites
Burial
Cremation
Death rites</td><td>165</td></tr>
</table>

		Online social networking<br>Internet<br>Social media<br>SARS-CoV-2<br>COVID-19	
Jan 31, 2021 8:00pm	**Social Work Abstracts (Ovid)**	**Database search does not utilize subject headings. Only identified key words were used.*	0
Jan 31, 2021 8:00pm	**Sociological Abstracts (ProQuest)**	Cremation<br>Death & dying<br>Funerals<br>Funeral industry<br>Funeral homes<br>Memorial services<br>Social networking web sites<br>Web apps<br>Webcasting<br>Webcasts<br>Web video<br>Websites<br>Web sites<br>World wide web<br>Internet<br>Streaming media<br>Social networks<br>Digital media<br>Web hosting<br>COVID<br>COVID-19<br>2019-nCoV<br>Coronavirus<br>Coronavirus disease 2019<br>Novel coronavirus<br>Social distancing<br>Coronavirus infections	35
Jan 31, 2021 8:00pm	**International Bibliography of Social Sciences (ProQuest)**	Cremation<br>Death & dying<br>Funerals<br>Funeral industry<br>Funeral homes<br>Memorial services<br>Social networking web sites<br>Web apps	62

		Webcasting<br>Webcasts<br>Web video<br>Websites<br>Web sites<br>World wide web<br>Internet<br>Streaming media<br>Social networks<br>Digital media<br>Web hosting<br>COVID<br>COVID-19<br>2019-nCoV<br>Coronavirus<br>Coronavirus disease 2019<br>Novel coronavirus<br>Social distancing<br>Coronavirus infections	
Jan 31, 2021 8:00pm	**Ageline (EBSCO)**	Funeral<br>Funeral arrangement<br>Funeral rites<br>Burial practice<br>Online<br>Internet<br>Social media<br>World Wide Web<br>Videoconferencing<br>Covid<br>Covid-19<br>2019-nCoV<br>Coronavirus<br>Coronavirus disease 2019<br>Novel coronavirus<br>Social distancing	16
Jan 31, 2021 8:00pm	**CINAHL (EBSCO)**	Funeral arrangement<br>Funeral rites<br>Burial practices<br>Online<br>Internet<br>Social media<br>World wide web<br>Videoconferencing<br>COVID	589

		COVID-19<br>2019-nCoV<br>Coronavirus<br>Coronavirus disease 2019<br>Novel coronavirus<br>Social distancing	
Feb 2, 2021 2:00pm	**Cochrane COVID-19 Study Register**	**Database search does not utilize subject headings. The search term was modified to include only "funeral"*	0
Feb 2, 2021 2:00pm	**World Health Organization COVID-19 Global Literature on Coronavirus Disease**	**Database search does not utilize subject headings. The search term was modified to include only "funeral"*	35
Feb 9, 2021 2:00pm	**Google Scholar**	*******The first 10 pages of google scholar were searched with the following modified key terms in order to fit within the constraints of the search functions of the database:* virtual funeral AND (covid-19 OR coronavirus OR covid)	100
Feb 9, 2021 3:00pm	**New York Academy of Medicine's Grey Literature Report**	**In order to fit within the constraints of the search functions of the database, the search terms were modified to include only the following key terms:* (online OR virtual) and (coronavirus OR covid OR covid-19) and (funeral OR memorial)	0
Feb 9, 2021 3:00pm	**OpenGrey**	**In order to fit within the constraints of the search functions of the database, the search terms were modified to include only the following key terms:* (online OR virtual) and (coronavirus OR covid OR covid-19) and (funeral OR memorial)	0

Feb 9, 2021 3:00pm	**US Newsstream Proquest**	**In order to fit within the constraints of the search functions of the database, the search terms were modified to include only the following key terms:* (online near/3 funeral*) AND (covid OR coronavirus OR pandemic)	483
Feb 9, 2021 3:00pm	**Canada Newsstream Proquest**	**In order to fit within the constraints of the search functions of the database, the search terms were modified to include only the following key terms:* (online near/3 funeral*) AND (covid OR coronavirus OR pandemic)	192
**Total:**			1795
**Total after duplicates removed:**			1,351
